# Evaluation of HPC Acceleration and Interconnect Technologies for High-Throughput Data Acquisition

**DOI:** 10.3390/s21227759

**Published:** 2021-11-22

**Authors:** Alessandro Cilardo

**Affiliations:** 1 Department of Electrical Engineering and Information Technologies, University of Naples Federico II, 80125 Naples, Italy; acilardo@unina.it; 2 CeRICT, 82100 Benevento, Italy

**Keywords:** HPC, interconnects, communication, FPGA, data acquisition

## Abstract

Efficient data movement in multi-node systems is a crucial issue at the crossroads of scientific computing, big data, and high-performance computing, impacting demanding data acquisition applications from high-energy physics to astronomy, where dedicated accelerators such as FPGA devices play a key role coupled with high-performance interconnect technologies. Building on the outcome of the RECIPE Horizon 2020 research project, this work evaluates the use of high-bandwidth interconnect standards, namely InfiniBand EDR and HDR, along with remote direct memory access functions for direct exposure of FPGA accelerator memory across a multi-node system. The prototype we present aims at avoiding dedicated network interfaces built in the FPGA accelerator itself, leaving most of the resources for user acceleration and supporting state-of-the-art interconnect technologies. We present the detail of the proposed system and a quantitative evaluation in terms of end-to-end bandwidth as concretely measured with a real-world FPGA-based multi-node HPC workload.

## 1. Introduction

Efficient data movement in multi-node systems is a crucial issue at the crossroads of related areas such as scientific data acquisition and computing, big data, and high-performance computing (HPC). This is strikingly confirmed by a plethora of previous works in the technical literature which point out the key role of high-performance interconnect technologies, e.g., for data acquisition, in demanding applications ranging from high-energy physics [1,2,3] to astronomy [4,5]. At the same time, these applications pose unprecedented processing requirements for the vast amount of data being generated and transferred, increasingly leading to the adoption of accelerator-based platforms, e.g., using graphics processing units (GPUs) or field-programmable gate arrays (FPGAs) in order to meet stringent energy-efficiency requirements through some form of customized computing. Consequently, many very recent works have explored advanced solutions for improving the end-to-end data communication performance targeting accelerator-based platforms, particularly those based on customized FPGA accelerators, in the context of scientific data acquisition and scientific computing [3,5,6,7,8,9].

Building on the outcome of the RECIPE Horizon 2020 research project, which aimed at the exploration of runtime system management HPC technologies, this work presents a prototype based on state-of-the-art high-performance interconnect technologies, namely InfiniBand EDR and HDR, and remote direct memory access (RDMA) functions for direct exposure of FPGA accelerator memory. As the main rationale behind the presented contribution, we evaluate the potential of high-end technologies available in HPC environments and their ability to address the convergence with the communication requirements of emerging big data applications. The presented solution was therefore defined with the following criteria in mind:

- Avoid dedicated network interfaces, but rather rely on standard infra- and inter-node interconnect technologies;
- Leave most of the space in the FPGA device for user acceleration, rather than using it for implementing the networking facilities;
- Follow the scaling of the interconnect technology, particularly InfiniBand, so that future performance will not be constrained by the custom FPGA implementation of the network interface.

This communication is structured as follows: Section 2 provides a brief introduction to the relevant technologies and an overview of recent works exploring efficient data movement in the context of accelerator-based big data/scientific computing applications. Section 3 describes the architecture of the proposed system. Section 4 presents an evaluation of the solution. Section 5 concludes the paper with a few final remarks.

## 2. Background and Key Technologies

As highlighted in the introductory section, numerous recent works point out the inherent need for high-performance data movement in big data/scientific computing applications, especially those based on custom acceleration. In fact, the development of an efficient infrastructure for data communication is a common problem addressed by the work plans of all major facilities and international projects supporting large-scale scientific experiments. For example, several works that have appeared in the literature in recent years involve interconnects for data communication in high-energy physics (HEP), e.g., for the Large Hadron Collider (LHC) at CERN [1,3], data acquisition from 2D X-ray detectors in the RASHPA platform at the European Synchrotron Radiation Facility (ESRF) [6], from gain adaptive detectors used for macromolecular crystallography [8], or from superheated emulsion detectors, e.g., based on FPGA multi-channel acquisition systems [9], as well as projects dealing with astronomic data, including the Square Kilometer Area (SKA) telescope [4] or specific applications such as adaptive optics [5].

For instance, a previous work used an FPGA device as a PCIe-based network interface card (NIC) for enabling RDMA-based GPU access [1], augmenting PCIe peer-to peer/RDMA capabilities borrowed from HPC technologies such as GPUDirect with a dedicated network stack, enabling predictable communication latency. The work in [5] shows that DMA between a custom FPGA-based frame-grabber and an accelerator/coprocessor across PCIe can meet the latency, throughput, energy efficiency and cost requirements of adaptive optics real-time computation for future extremely large telescopes (ELTs). Aimed at supporting Ethernet, InfiniBand, and similar network fabrics interchangeably, the contribution in [3] introduces an asynchronous message service, demonstrated in a laboratory environment with Ethernet and FDR InfiniBand networks for the ATLAS experiment at LHC [2], supporting high-level programming models used in workloads of interest for high-energy physics applications. The authors of [6] address the problem of acquiring data from 2D X-ray detectors (either images, regions of interest, metadata, or events) and delivering them to multiple backend computers by developing an RDMA over Ethernet protocol and designing a 100 G MAC component for Xilinx UltraScale+ FPGAs and then comparing the dedicated link with a standard RDMA over Converged Ethernet v2 (RoCEv2) protocol using commercial Mellanox adapters. Based on this, the work in [7] implements a real-time FPGA-based image manipulation system used in a backend board of a photon-counting detector as part of the RASHPA data acquisition platform at ESRF. The contribution in [8] addresses macromolecular crystallography, the dominant method for high-resolution structure determination of biomolecules, and specifically, it uses raw Ethernet (or zero-copy) transfers for low-latency data acquisition from gain adaptive detectors, which can be accomplished without the CPU in the receiving machines being involved in the transfers themselves. While based on RDMA, unlike the case of [6], the sending machine in [8], i.e., the detector, is not aware of RDMA as it just handles standard UDP/IP packets, while the receiver uses the RDMA API. The authors of [10] propose standalone FPGAs to be used as network-attached accelerators as a possible alternative to PCIe-attached devices. They present a network stack supporting a reduced version of RoCEv2 as well as InfiniBand or UDP/IP communication, reaching competitive results in terms of bandwidth and latency. The contribution in [11] discusses the state of FPGA acceleration in HPC, with particular emphasis on the impact of the underlying interconnect, and proposes a hardware-offloaded transport supporting a global memory space for distributed FPGA accelerator access, achieving latency reductions versus a software-based transport and improved computing throughput for an example HPC workload. The work in [12] compares the network performance of FPGAs against bare-metal servers, virtual machines, and Linux containers, finding improved round trip times and throughput levels for the case of FPGAs. The authors of [9] present a dedicated FPGA-based multi-channel data acquisition system for superheated emulsion detectors developed as a standalone, portable component to be connected to a PC through a UART link.

Note that many of the above works inherently rely on fully dedicated FPGA-implemented support for handling network tasks, resulting in reduced acceleration resources available for user logic, a need for custom physical links to be deployed in the facility for direct accelerator connection, and impossibility to exploit commercial high-performance adapters, e.g., InfiniBand cards, which guarantee the maximum degree of performance and optimization for network processing.

### Key Technologies

InfiniBand [13] is a networking standard targeted at high-performance interconnects, particularly those deployed in high-end datacenter and supercomputing facilities. It supports direct or switched interconnection between computers and dedicated nodes such as memory, storage, and possibly acceleration nodes. InfiniBand relies on a switched fabric network topology and is designed with scalability requirements in mind. In fact, InfiniBand is one of the leading interconnect technologies used in top HPC systems, competing with alternatives which include Ethernet and Intel Omni-Path. The InfiniBand Trade Association [13] promotes the evolution of the technology, which is available in several families including single-data rate, double-data rate, and quad-data rate signaling. State-of-the-art products that are commercially available at the time of writing support the HDR standard, providing a signaling rate of 50 Gbit/s and an aggregated bandwidth of 200 Gbit/s for a typical four-link connection.

Among other features, InfiniBand was developed to directly support remote direct memory access (RDMA), which allows a memory region in a host system to be accessed from a remote node without the host operating system being involved in the transfer, thus substantially offloading networking processing overheads from the host CPU and only entrusting the network adapter to handle data movement within the accessed host. By eliminating the need for memory copy operations between the user application memory and the operating system data buffers, RDMA effectively supports zero-copy networking and can reduce transfer latencies and improve throughput levels significantly. The model enabled by RDMA is inherently based on single-sided communication, as the accessed node receives no notification of the transfer completion. The RDMA API was originally supported by InfiniBand, but consolidated implementations of RDMA are available today for Ethernet, namely RDMA over Converged Ethernet (RoCE), iWARP, Omni-Path, and virtual interface architecture, in addition to InfiniBand itself. RDMA is supported natively by the Linux and Windows operating systems, while additional support is also available for other systems.

Of course, RDMA performance critically depends on the internal infrastructure available for data transfers in the target host system, the PCIe interconnect in most cases. A feature of special relevance is PCIe peer-to-peer (P2P) communication, enabling two PCIe devices to directly transfer data between each other, including the particular case when one of the two devices is a network adapter with RDMA capabilities, as well as situations where multiple components of the host system, e.g., different storage and acceleration cards, need to exchange data. Note that the physical PCIe interconnect can be configured by the host system manufacturer in different topologies, which may variously impact the effective bandwidth achieved with PCIe P2P communication, as pointed out in Section 4.

## 3. System Architecture

This work evaluates and compares two different system configurations, focusing on the impact of a few key design choices. The first configuration, which we refer to as baseline, was developed in the context of the Horizon 2020 research project RECIPE, aimed at the exploration of runtime system management HPC technologies. The second configuration, referred to as the backplane-based system, extends and improves the baseline solution. For both systems, we were particularly interested in evaluating the possibility of exploiting state-of-the-art InfiniBand interconnect technology along with RDMA for direct access to FPGA-based accelerators across multiple nodes, without resorting to custom FPGA-implemented network interfaces and achieving near peak throughput levels. The baseline configuration developed within the scope of the project comprises, among other hardware, two HPC-class servers, namely two 1U Supermicro SuperServer 1029GQ-TNRT machines [14], able to host up to four GPU/FPGA acceleration cards through four PCI-Express 3.0 ×16 full-height full-length (FHFL) slots. The two machines are each equipped with a Mellanox MCX555A-ECAT DDR network card [15] and connected to each other through an EDR InfiniBand link. In addition, a Xilinx Alveo U280 card is installed in one of the two machines, featuring a large HPC-grade UltraScale+ device, 8 GB of integrated HBM2 offering an aggregated bandwidth (over 32 banks) of up to 460 GB/s, 32 GB of DDR4 memory, a PCIe Gen3 ×16 (or Gen4 ×8) interface, and two QSFP28 connectors for 100 GB/s Ethernet links.

**Figure 1:**
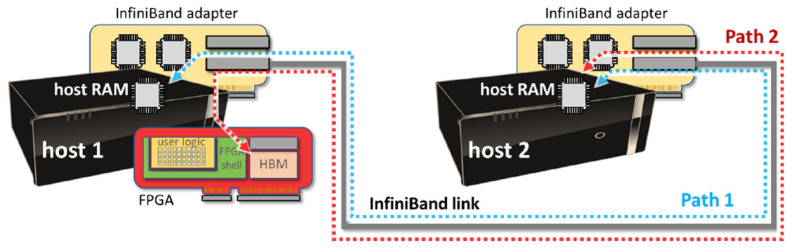
Schematic of the baseline system. The dashed arrows indicate the communication paths exercised in our experimental evaluation.

In the context of the project, we implemented a custom FPGA hardware runtime, or FPGA shell, i.e., the statically configured FPGA region hosting at least a PCIe interface block and responsible for programming the dynamically reconfigurable regions with user-specified kernels. In addition to handling partial reconfiguration, the FPGA shell is also used to: (1) expose a set of control/monitoring mechanisms to the host-side resource management system, e.g., enabling the collection of fine-grain physical parameters and accelerator-specific performance counters, (2) support hardware-level transparent checkpointing/restart functions (not covered by this paper), and (3) enable intra- and inter-node communication primitives for effective integration of the device in large-scale multi-node HPC applications. In particular, the FPGA shell we developed features a customized PCIe controller, based on the Xilinx XDMA IP component, which significantly impacts local/remote communication capabilities, including PCIe peer-to-peer communication and remote direct accelerator memory access through the high-performance InfiniBand adapter. In fact, the FPGA resources, such as card memory and any memory-mapped FPGA component, can be accessed through the PCIe bus by means of the XDMA driver and the associated hardware component. Although the XDMA component exposes several interfaces for integration with the host (i.e., AXI Memory Mapped interface, AXI Bypass interface, AXI Stream interface, and AXI Lite interface, the first two being potentially relevant for our work), we disabled the DMA capabilities of the XDMA IP so that the Xilinx XDMA drivers are not used at all and the interaction with the card does not rely on the XDMA device files. Technically, this is achieved by enabling the AXI Bypass interface on the XDMA, which lets the device memory be directly exposed for PCIe peer-to-peer and RDMA interactions (controlled by the network adapter), without the host CPU being aware of the communication. The configuration results in the FPGA card exposing three PCIe memory regions through three base address registers (BARs): BAR0 used for control operations, BAR2 for DMA operations, and BAR4 for RDMA. Figure 1 shows a schematic of the implemented system.

As mentioned above, in this work we also evaluate an evolved configuration built as a follow-up activity of the RECIPE project. The key motivation for the evolution of the initial setup is that commercial off-the-shelf machines, such as those we used, are often based on PCIe configurations which are not ideal for peer-to-peer communication between the network adapter and the acceleration device, since they make PCIe communications traverse an interconnect between PCIe host bridges within a root complex in a CPU or traverse a CPU interconnect such as QPI/UPI. The impact of this setup will be quantitatively presented in Section 4. The backplane-based system configuration was driven by the above observation. It features a single on-board PCIe switch directly connecting the communicating devices, particularly the high-performance network adapter and the FPGA card, thereby providing the highest PCIe bandwidth for peer-to-peer interaction. Complying with the requirements identified in Section 1, we aimed at preserving the use of commercial HPC-class machines, network interface cards, and interconnect technologies. As a consequence, the extended configuration just relies on a standard machine along with an extension subsystem made of a custom-assembled PCIe gen4 backplane hosting a ConnectX-6 VPI 200 GB/s InfiniBand card by Mellanox, along with the Xilinx Alveo U280 card (which is a PCIe gen3 ×16 card, thereby still limiting the peak theoretical bandwidth to 16 GB/s), offering the ideal setting for PCIe peer-to-peer communication. More specifically, the setup relies on a One Stop Systems model-522 PCIe expansion backplane featuring five PCIe ×16 slots and mounting a Broadcom PEX88096 98-lane, 98-port, PCI Express Gen 4.0 switch, which supports up to 48 DMA channels. Powered by an embedded ARM Cortex R4 CPU, the Broadcom switch enables I/O sharing with standard SR-IOV or multifunction capability, allowing multiple hosts to reside on a single PCIe topology. Hosts communicate through generic DMA or NT with other standard hosts and end-points using application software. Additionally, the switch features purpose-built support for NVMe all-flash array (AFA) systems. The backplane-based system we developed also includes a PCIe carrier card equipped with four 2TB SSD devices, totaling 8TB of storage available within the backplane, and can possibly be used to host newly introduced PCIe-based computational storage drives augmented with reconfigurable hardware accelerators [16].

**Figure 2:**
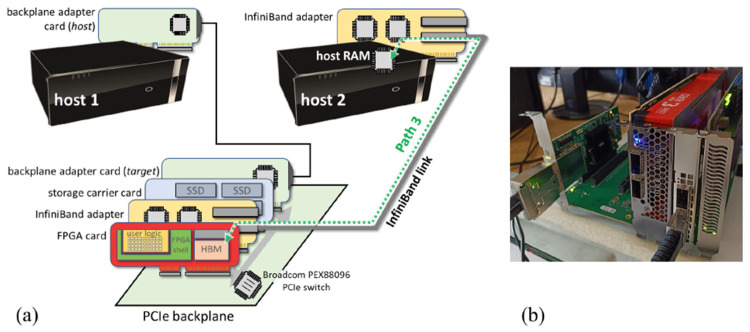
(**a**) Schematic of the backplane-based system. The dashed arrow indicates the communication path exercised in our experimental evaluation. (**b**) Photograph of the system.

Figure 2a shows a schematic of the implemented system relying on the dedicated PCIe backplane extension, while Figure 2b shows a photograph of the physical prototype. The system can be readily plugged in a standard setting and is seen as a normal tree of PCIe devices, while no dedicated cabling or network interfaces and links are required in the physical setup, apart from the local cable between the host and the backplane. The system extension can be easily fit into a chassis (being designed at the time of writing), featuring a standard form factor for direct mounting in normal rack enclosures.

## 4. Evaluation

We were interested in comparing the real-world end-to-end throughput levels achieved by RDMA communication as concretely measured in the prototype with the theoretical peak compute and communication rates. We evaluated the setup by means of both single transfers of various sizes and a real-world application handling a large dataset.

**Figure 3:**
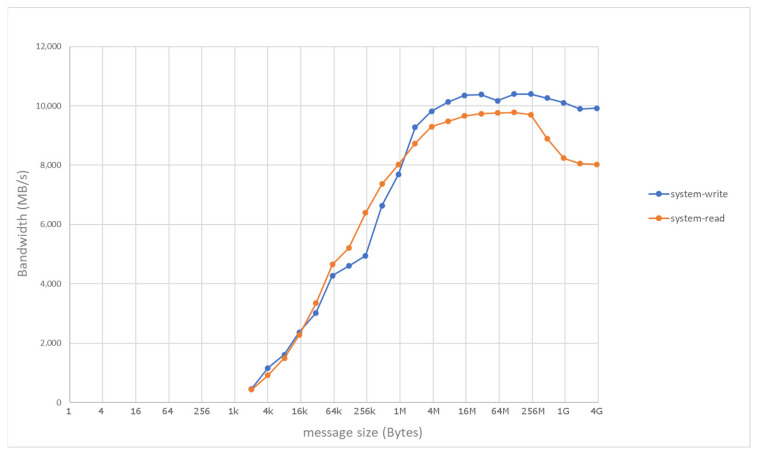
Host-to-host RDMA performance, as measured in the baseline system along Path 1.

**Figure 4:**
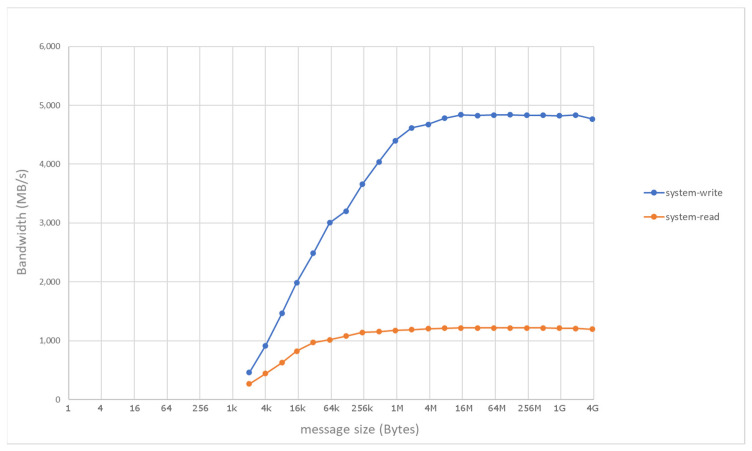
Performance of remote direct accelerator memory access based on PCIe peer-to-peer communication, as measured in the baseline system along Path 2.

Considering the baseline configuration, Figure 3 shows the communication performance of RDMA as measured by means of manufacturer-provided test utilities along Path 1 in Figure 1, i.e., only involving the main memory of the two hosts. In the baseline setup, the performance closely approaches theoretical peak values of the InfiniBand link. On the other hand, Figure 4 shows the numbers achieved with RDMA and PCIe peer-to-peer communication along Path 2 in Figure 1, i.e., direct write/read operations to/from the HBM in the FPGA card. The measured communication rates are in the same order of magnitude of the theoretical values, particularly for remote write operations (measured bandwidth of around 4.8 GB/s), while remote read operations experience a higher penalty (around 1.2 GB/s).

As a real-world case-study application, we implemented a stencil kernel, which is common in many HPC applications, including computer simulations for scientific and engineering applications, e.g., for fluid dynamics, partial differential equations, cellular automata, image processing, etc. Generic iterative stencil codes work by sweeping a multi-dimensional data structure, typically a two- or three-dimension grid in each iteration by using a fixed-size window, updating the whole structure for the next iteration. The code is representative of computation kernels and memory access patterns that are typically found in HPC and is suitable for FPGA acceleration [17]. The RDMA communication mechanisms available for our custom FPGA shell provide an optimal fit for such patterns. We developed an FPGA accelerator handling the computation and data access for a demo stencil application, particularly a five-point Jacobi stencil, matching the potential of dedicated acceleration and the HBM technology for local (on card) communication, based on a stream-oriented design which sustains a computation throughput of around 7.2 GB/s and hence fully exploits the bandwidth of one of the HBM banks. We parallelized the application assuming a multi-node scenario where each acceleration node is assigned a tile of the large matrix processed by the overall stencil application. Tiling is performed row-wise and each tile, having a size in the order of gigabytes of memory, is transferred by means of RDMA mechanisms to the accelerator HBM. Standard parallelization practices have been adopted for the stencil application, including halo regions redundantly transferred with each tile to the acceleration nodes.

**Figure 5:**
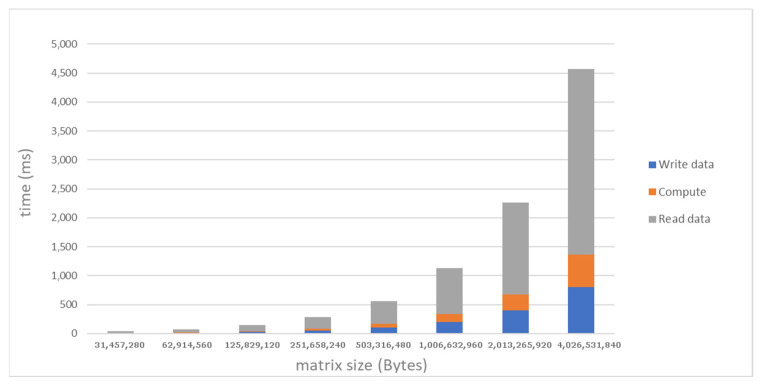
Performance of the stencil case study with remote direct accelerator memory access based on PCIe peer-to-peer communication, as measured in the baseline system along Path 2.

The baseline system was also used to collect quantitative measurements for the stencil use case. Figure 5 shows the breakdown of the communication and execution time for various sizes of the matrix processed by the stencil code, as measured in the baseline system along Path 2. The levels of communication and computation throughput are in the same order of magnitude and, overall, for a 4 GB tile the transfers to/from the remote node through RDMA and the FPGA stencil computation require a total time of around 4.57 s. As in the case of single transfers, we noted nonideal bandwidth values and asymmetry in read operations. These effects are inherently related with the PCIe configuration in the underlying system hosting the FPGA card, including low-level factors such as PCIe bus transactions and the proprietary details of the network adapter architecture [18]. In our case study, they inevitably prevent the full exploitation of the maximum compute rate featured by the stencil accelerator, i.e., 7.2 GB/s.

**Figure 6:**
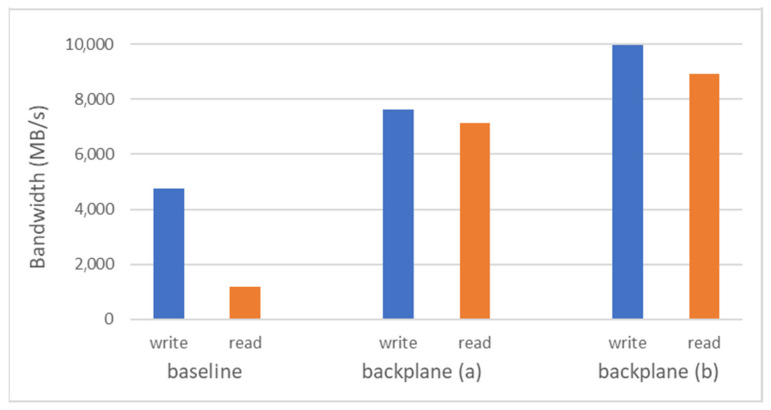
Comparisons of the performance of remote direct accelerator memory access based on PCIe peer-to-peer communication in the case of the baseline and the backplane-based system. The backplane cases (**a**) and (**b**) differ in the internal HBM interface clock frequency, 250 MHz and 450 MHz, respectively.

For the backplane-based system we performed similar tests, but exercising Path 3 in the extended prototype architecture of Figure 2a. We observed similar trends as the message size increased, but with significantly improved absolute values compared to the baseline system, around 10 GB/s for write transactions and 8.9 GB/s for read transactions, which is remarkably closer to the theoretical values. As a last experiment, we were interested in evaluating the impact of on-chip memory interfaces on the collected numbers. The on-chip interface to the HBM fabric relies on an FPGA-implemented controller by Xilinx which is optimally run at 450 MHz. For our evaluation, we lowered the clock frequency to 250 MHz, a second value that is supported by the HBM controller core. With this setting, the maximum bandwidth (for communication only) turned out to be around 7.6 GB/s for write transactions and 7.1 GB/s for read transactions. This finding points out that, as conjectured, the PCIe topology remains the major bottleneck potentially affecting the exploitation of the peak bandwidth, while the internal HBM interface speed has a moderate (but non-negligible) impact. The results and comparisons are summarized in Figure 6.

The key takeaway from the above experiments is that by adopting a carefully designed system architecture, the bandwidth results for end-to-end transfers of large data buffers are close to the theoretical peak bandwidth and similar to state-of-the-art works, which do, however, rely on custom FPGA-implemented network interfaces [10].

Once again, note that the backplane-based system uses commercial-off-the-shelf products and can be easily deployed in a high-performance computing facility, as it is just a PCIe-based extension of standard machines and exposes standard interfaces, particularly to an InfiniBand interconnect. Furthermore, note that some commercial servers, for example the SuperMicro products targeted at GPU acceleration (SuperServer 1029GQ-TNRT models [19], belonging to the same product line as the servers we acquired), feature dedicated PCIe switches serving multiple acceleration and network cards, enabling optimized topologies for peer-to-peer communication. Such setups can potentially provide similar results to our FPGA shell in the backplane-based system, further pushing the adoption of commercial-off-the-shelf, high-performance interconnect technologies for data communication in FPGA-based HPC settings. The proposed backplane-based system provides, however, a much more cost-effective solution than the above high-end products. In addition, as already highlighted before, the backplane-based system can be readily used for hosting newly introduced computational storage drives such as Xilinx SmartSSD. Interestingly, such devices are in turn internally based on a dedicated PCIe switch [16] for improved peer-to-peer communication. The backplane system would potentially serve as a second level in a hierarchical PCIe topology, possibly connecting together multiple computational storage cards with a high-performance network interface, as shown in this work.

## 5. Conclusions

FPGA-based acceleration and efficient communication is critical for data acquisition applications in high-performance settings. While a number of recent solutions address the problem by relying on custom FPGA-implemented network interfaces, our work evaluated the use of high-bandwidth interconnect adapters, namely Mellanox adapters supporting InfiniBand EDR and HDR standards, along with RDMA for direct exposure of FPGA accelerator memory across a multi-node system. Avoiding dedicated network interfaces, our system leaves most of the FPGA resources for user acceleration. As pointed out by the experimental evaluation, the system reaches near peak end-to-end communication throughputs as measured with a real-world application. As key strengths, the solution we propose only relies on commercial-off-the-shelf technologies, can be readily plugged in a standard setting, and requires no dedicated cabling or network interfaces and links. Furthermore, supporting standard high-performance interconnect technologies, the system can potentially follow their scaling towards higher bandwidth levels in the near future.

## Data Availability

Not applicable.
